# Acute enhancement of Romanian deadlift performance after consumption of caffeinated chewing gum

**DOI:** 10.1038/s41598-023-49453-y

**Published:** 2023-12-12

**Authors:** Chun-Hung Chen, Shih-Hao Wu, Yi-Jie Shiu, Sheng-Yan Yu, Chih-Hui Chiu

**Affiliations:** 1 Department of Emergency Medicine, China Medical University Hospital, China Medical University, Taichung, Taiwan; 2 College of Medicine, China Medical University, Taichung, 404 Taiwan; 3 Division of Toxicology, China Medical University Hospital, China Medical University, Taichung, Taiwan; 4 Graduate Program in Department of Exercise Health Science, National Taiwan University of Sport, No. 16, Sec. 1, Shuang-Shih Rd., Taichung, 404 Taiwan; 5 Department of Physical Education and Sport Sciences, National Taiwan Normal University, Taipei, Taiwan

**Keywords:** Physiology, Metabolism

## Abstract

This study investigates the effect of the consumption of caffeinated chewing gum on the performance of Romanian deadlift on the flywheel training device. A total of 19 participants completed a randomized, cross-over, single-blind experiment with food-grade caffeinated chewing gum trial (CAF) or placebo trail (PL). Gum were chewed for 10 min and rest for 15 min prior to the Romanian deadlift test using the inertial resistance training machine. 5 sets of 6 Romanian deadlifts were performed, with a 3-min rest between sets, followed by a 7-day washout period before the next set of trials. The average power, average force, total peak power, peak concentric power, peak eccentric power, heart rate, and rating of perceived exertion (RPE) for each trials were analyzed using paired-T test. Compared to placebo, caffeinated chewing gum trial enhanced peak concentric power (*P* = 0.016, Cohen's d = 0.44), peak eccentric power (*P* = 0.005, Cohen's d = 0.55), average power (*P* = 0.013, Cohen's d = 0.43), and total work (*P* = 0.026, Cohen's d = 0.28). However, in average force (*P* = 0.063, Cohen's d = 0.50), RPE (*P* = 0.266), and heart rate (*P* = 0.143), were no significant differences between trials. Caffeinated chewing gum with a dose of caffeine of 200 mg for 10 min may acutely enhance Romanian deadlift performance on the flywheel machine.

## Introduction

In recent years, resistance training has become increasingly popular. Training methods typically involve lifting some sort of weight, such as barbells, dumbbells, and pulleys. This type of training focuses primarily on counteracting the force of gravity. In contrast, flywheel training, also known as inertial training, uses the inertia of the flywheel to provide resistance during the concentric (muscle shortening) and eccentric (muscle lengthening) phases of movement. Recent literature using inertial flywheel resistance training has shown that strength, power and performance, such as the vertical jump and the first 5 m of the sprint test, are usually significantly improved after 4–8 weeks of flywheel training^1–4^. A meta-analysis of 20 experimental studies found that flywheel training resulted in statistically significant increases in muscle hypertrophy, maximal dynamic strength, power, and horizontal and vertical movement^5^. Studies also indicated that explosive force generation is highly related to muscle strength and athletic performance^6,7^. Therefore, concentric and eccentric training is particularly important in training.

Caffeine consumption is widespread, with approximately 90% of adults consuming some form of caffeine in their diet and approximately 30–60% of athletes consuming caffeine as a sports supplement^8^. The available literature suggests that caffeine supplementation may improve aerobic and anaerobic exercise performance^9–11^. The absorption rate of caffeine is significantly accelerated by chewing gum, and the concentration of caffeine in the blood can quickly reach a peak in about 5–10 min, which can improve sports performance and mental state^12^. Therefore, it is possible to avoid taking solid or liquid supplements during intense exercise to maintain exercise performance and reduce the risk of gastrointestinal discomfort.

However, it is still unclear whether caffeinated chewing gum supplementation can improve the performance of the Romanian deadlift on a flywheel. Therefore, the aim of this study was to investigate the effect of a caffeinated chewing gum supplementation on the performance of the Romanian deadlift on a flywheel training device.

## Materials and methods

### Experimental design

**Figure 1:**
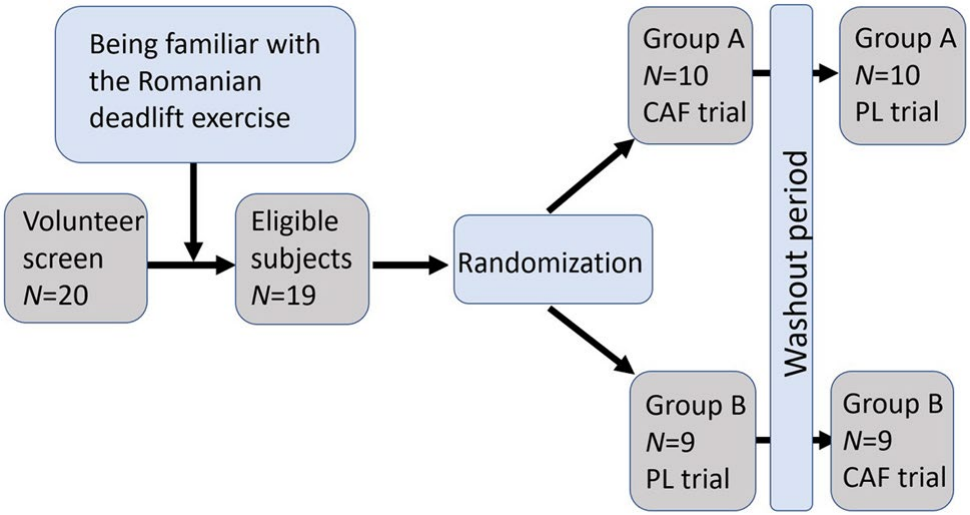
CONSORT diagram and study design.

The study was a randomized, crossover, single-blind study design in which participants were randomized to ingest a caffeinated chewing gum trial (CAF) and a placebo gum trial (PL) 7 days apart, 10 min before performing the Romanian deadlift on a flywheel training device, CONSORT diagram and study design as shown in Fig. 1. We used computerized randomization using Excel Office 365 to arrange the order of participants in the experiment. Caffeine was administered at an absolute dose of 200 mg (Military Energy Gum®, Ford Gum and Machine Go, Akron, NY, USA). The placebo was a similar looking and tasting gum that did not contain caffeine (xylitol, lime mint, green; Lotte, Saitama, Japan). The participants chewed the gum for 10 min and then spat it out into a container. The exercise session started immediately afterwards.

The participants made three visits to the laboratory. During the first visit, participants were familiarized with the exercise tests and the maximum resistance was determined. Following the familiarization visit, participants were randomized in a counterbalanced fashion to two main conditions, PL and CAF. The primary outcome was the performance of the Romanian deadlift on a flywheel training device, and the secondary outcomes were heart rate and RPE.

### Participants

All 19 participants (age 22.5 (3.5) years; height 176.2 (6.5) cm; body mass 78.8 (13.2) kg; habitual caffeine intake: 62.55 (94.01) mg/day). A priori power analysis, using an expected effect size (f) of 0.55 for peak eccentric power on the Romanian deadlift test, an α of 0.05, a statistical power of 0.80^13^, indicated that the minimum sample size for this study was 19 participants. These participants met the inclusion criteria of having regular exercise habits, with 3–5 days per week and at least 30 min per session; having experience with strength training; and being familiar with the Romanian deadlift exercise. They were excluded if they were: (1) individuals with certain medical conditions such as hypertension, diabetes, and kidney disease; (2) any sports injuries in the previous six months, such as ligament tears or inflammation and tendon ruptures in the knee joint, strains of the biceps or quadriceps muscles, or strains of the biceps muscle.

All participants were interviewed verbally about their exercise habits over the previous 6 months. They were instructed to avoid caffeine-containing foods such as coffee, energy drinks, tea and chocolate for 24 h prior to the formal experiment. Excessive exercise training was prohibited for 72 h prior to the study. All potential problems that participants may encounter during the experiment were explained before the study began and the procedure was fully explained. Participants were asked to complete an informed consent form. This study was approved by the Institutional Review Board of Jen-Ai Hospital—Dali Branch (111-09) and registered in the ClinicalTrials.gov (Date: 22/02/2022; ID “NCT05900349”; https://register.clinicaltrials.gov). This study follows the principles of the Declaration of Helsinki and follows the recommendations proposed by the CONSORT Statement.

### Experimental protocol

**Figure 2:**
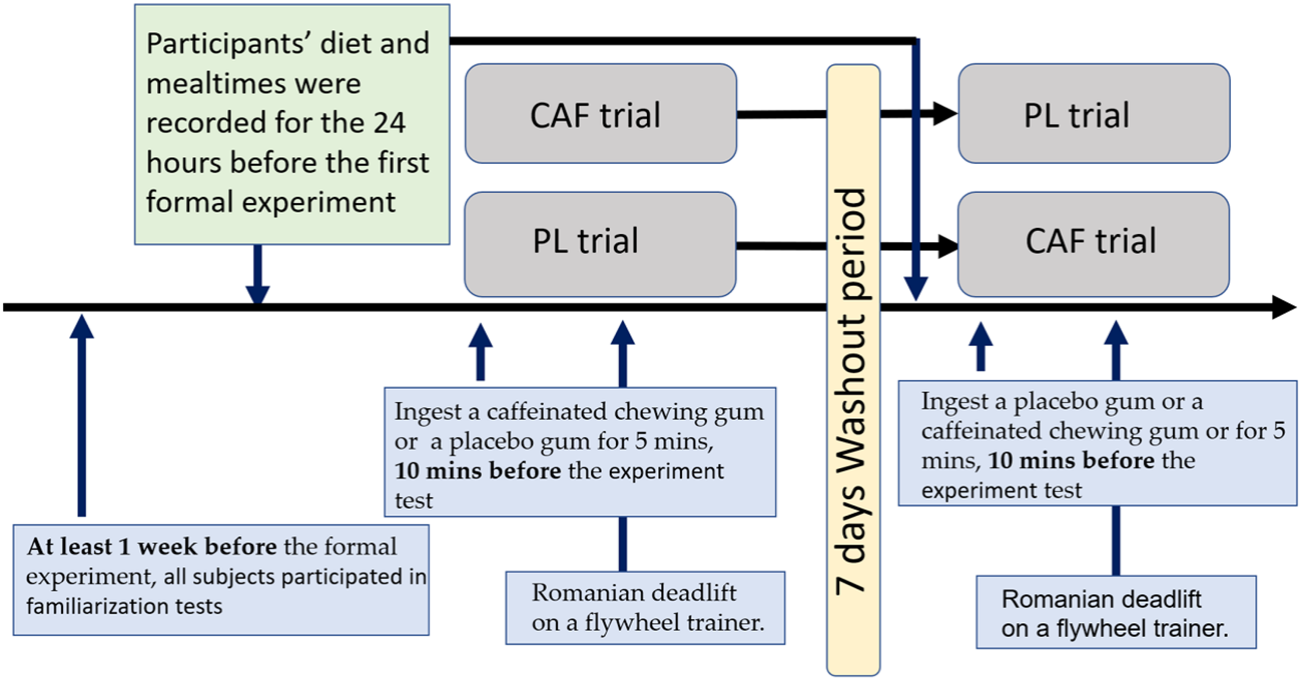
Experimental protocol.

Experimental protocol was shown on Fig. 2. This study consisted of two phases: a preparation phase and a formal experimental phase. Prior to the formal experiment, all participants were required to complete the preparation phase, during which they familiarized themselves with the operation of the experimental apparatus and practiced the experimental procedures. A pre-test was conducted to determine the maximum resistance that each participant could handle by trying different resistances (i.e., 0.050, 0.075, and 0.100 kg/m^2^). The resistance at which the participant achieved maximum strength was recorded and used for the formal experiment.

On the day before the first formal experiment, the participants' dietary intake was recorded, and they were asked to replicate the same diet before the next experiment. Participants were also instructed to avoid consuming caffeinated foods for 24 h before the formal experiment and to refrain from excessive exercise training for 72 h.

On the day of the experiment, the participants arrived at the laboratory in the afternoon, following their regular training schedule. After a 5-min warm-up on a bicycle ergometer, they chewed either food-grade caffeinated chewing gum (200 mg per serving) or a placebo gum for 5 min. After a 10-min rest period, participants performed a set of 6 repetitions and 5 sets of Romanian Deadlift (RDL) exercises using the non-gravity-dependent flywheel inertial device (K-Box 4, Exxentric®, Stockholm, Sweden). There was a 3-min rest period between sets. The Exxentric kMeter (© Exxentric AB, Sweden), connected to its application via Bluetooth, was used in each RDL test to record average power (W), average force (N), peak concentric power (W), peak eccentric power (W) and total work (KJ). This device had been used in other studies and had demonstrated good reliability and validity in measuring various performance indicators^14^. Participants wore wrist-based heart rate monitors to track their heart rate in beats per minute (bpm) in real time. These non-intrusive devices provided accurate cardiovascular data during physical activity. At the same time, participants rated their exertion using the Rating of Perceived Exertion (RPE) scale, which ranges from 6 (no exertion) to 20 (maximum exertion). This approach allowed us to correlate objective heart rate measurements with subjective exertion ratings, providing a comprehensive assessment of the physical demands experienced by the participants. In the CAF trial, 8 out of 19 (42%) participants incorrectly guessed whether the gum contained caffeine. In the PL trial, 11 out of 19 (57%) participants incorrectly guessed whether the gum contained caffeine.

After completion of the first experiment, participants had a 7-day rest and washout period before proceeding to the next set of experiments. All trials were completed within one month, followed by analysis of the experimental results.

### Statistical analysis

The Shapiro–Wilk test was conducted to test the normality of the data. Two-way ANOVA was used to compare the differences in heart rate, RPE and other measures between the CAF trial and the PL trial before and after the Romanian deadlift inertia resistance training test. We represent standard deviations in parentheses [i.e., mean (SD)]^15^. For the 6 repetitions, the average of the last 5 repetitions was determined by removing the first accelerated repetition. Paired samples t-tests were used to compare average power, average force, peak concentric power, peak eccentric power and total work of the five groups. To quantify the magnitude of the effects observed in this study, the effect size was calculated using Cohen's d formula. Including Cohen's d in our analysis helps to understand the practical significance of the results, and complements the statistical significance reported. The significance level was set at α < 0.05.

### Ethics approval and consent to participate

This study was approved by the Institutional Review Board of Jen-Ai Hospital (111-09) in Taiwan.

## Results

All 19 participants completed the test sessions and were included in the analysis. According to the pre-test dietary questionnaire, the participants had an average daily caffeine intake of 62.55 (94.01) mg. The absolute dose of caffeine given in this experiment was 200 mg, and after weight calculation, the caffeine intake of each participants was 2.75 (0.53) mg/kg. Based on the pre-test results, the flywheel resistance during the RDL exercise was 0.06 (0.02) kg/m^2^ and the average power was 378.2 (146.7) W.

**Figure 3:**
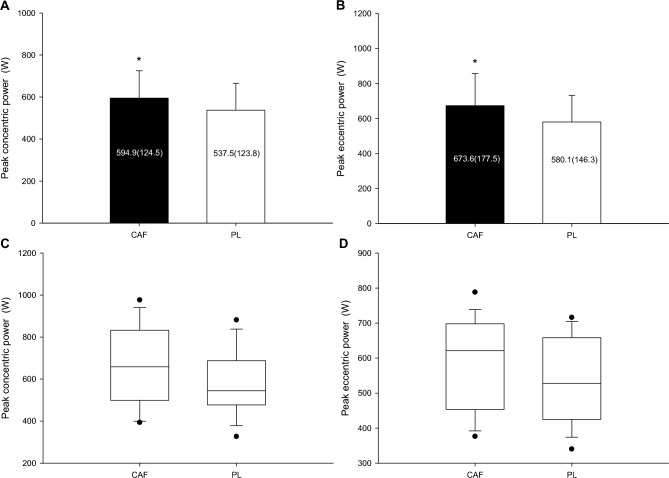
Peak concentric and eccentric power. The peak concentric power (**A**) and peak eccentric power (**B**) of the CAF and PL groups were compared. *CAF was significantly higher than those for the PL. The boxplot of each plot is displayed as (**C**, **D**).

Comparison of peak concentric power (Fig. 3A) between the two trials revealed a higher peak in the CAF trial compared to the PL trial, with a statistically notable difference (P = 0.016, Cohen's d = 0.44). When assessing peak eccentric power (Fig. 3B), the analysis indicated that the CAF trial had a higher peak eccentric power relative to the PL trial, with a statistically noticeable difference (P = 0.005, Cohen's d = 0.55). The boxplot of each plot is displayed as Fig. 3C,D.

**Figure 4:**
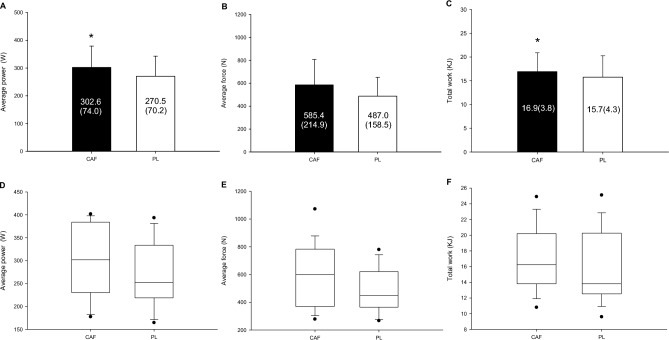
The average power (**A**), average force (**B**), and total work (**C**) of the CAF and PL trials were compared. *CAF was significantly higher than those for the PL. The boxplot of each plot is displayed as (**D**–**F**).

Figure 4A presents the comparison of mean power between the CAF and PL trials. The analysis suggests a higher average power in the CAF trial compared to the PL trial, with a statistically remarkable difference (P = 0.013, Cohen's d = 0.43). For mean strength (Fig. 4B), the two trials demonstrated a non-substantial difference in results (P = 0.063, Cohen's d = 0.50). In addition, total workload (Fig. 4C) was higher in the CAF study compared to the PL study, which was reflected in a statistically notable difference (P = 0.026, Cohen's d = 0.28). The boxplot of each plot is displayed as Fig. 4D–F.

**Figure 5:**
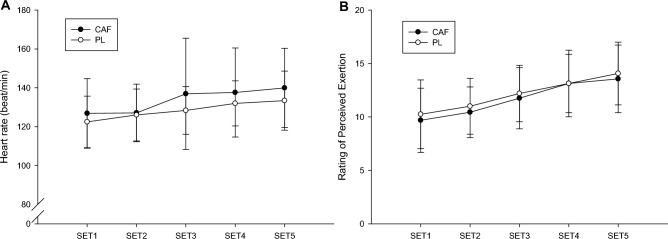
Comparison of heart rate and rating of perceived exertion between the CAF and PL trials.

Participants' heart rates were monitored and RPE levels were assessed (Fig. 5). Analysis revealed no statistically significant difference in heart rate across the five trials (trial time = 0.440, trial = 0.151, time < 0.001). Similarly, the comparison of mean heart rate between the two trials (CAF: 132.9 (18.7) bpm; PL: 127.9 (12.7) bpm; p = 0.143) showed no discernible difference. For RPE, the data across the five trials did not show a pronounced difference (trial time = 0.634, trial = 0.282, time < 0.001) and the mean RPE between the two trials (CAF: 11.7(2.4); PL: 12.1(2.5); p = 0.266) did not differ markedly.

## Discussion

The main findings of this study were that caffeine ingestion via caffeinated chewing gum significantly improved the performance of the Romanian deadlift on the flywheel exercise compared to placebo in peak concentric power, peak eccentric power, average power and total work. However, there were no significant differences between the two trials in average force, RPE and heart rate.

In previous studies, caffeine was mainly ingested in the form of capsules, which took approximately 45–60 min to reach high concentrations in the blood^16^. However, in recent years, many studies have explored the use of caffeine-containing chewing gum as an alternative method of ingestion^12,13^. Studies have found that caffeinated chewing gum absorbs caffeine at a significantly faster rate than capsules^12^. When chewing caffeinated gum, absorption takes place mainly through the mucous membranes in the mouth. Due to the rich blood supply in the mouth, caffeine can be absorbed quickly and works in a short time^17^. With caffeinated chewing gums, the initial increase in plasma caffeine concentration occurred within ten minutes post-ingestion^12^. In a study by Morris et al., 15 participants chewed 200 mg of caffeinated gum for 10 min and found that 96.2% of the caffeine in the gum was absorbed into the blood circulation of the participants^18^. The caffeinated gum used in this study was of the same brand as in this study, and the duration of chewing and the rest period after chewing were in accordance with the recommended values in this study. Therefore, in our protocol, the participants chewed the gum for 10 min and then spat it out into a container.

Lopes-Silva has demonstrated that caffeine intake can improve intermittent upper body strength endurance performance in combat sports athletes^19^. Ferreira has also indicated that caffeine had a significant facilitating effect on muscular endurance and maximum strength in the bench press^20^. Caffeine supplementation has been proved to improve physical performance in muscular strength and endurance, endurance exercise testing, jumping and sport specific movements^5,21^. Although there is moderate to high-quality evidence from systematic reviews that caffeine has a facilitative effect on various types of exercise^22^. The present study supports the findings of these studies and confirms that the use of caffeinated chewing gum is effective in enhancing RDL performance on a flywheel training device.

In the recent literature, only one study was found in which inertial flywheel resistance performance was measured with 6 mg/kg caffeine supplementation, and the study found significant improvements in total mean and peak power, and centripetal and centrifugal mean and peak power^23^. The present study used a fixed dose of 200 mg of caffeinated chewing gum intervention, and the results were similar even though the measured movements and doses were different. Since the inertial flywheel resistance machine is a high-intensity exercise mode, it can be investigated from other high-intensity resistance exercise studies. Caffeine significantly enhanced lower extremity strength with no difference in response to higher doses, whereas upper extremity strength was only enhanced at doses of 4 and 6 mg/kg^24^. In addition, research has shown that caffeine intake enhances muscular endurance, speed, power, isometric and isokinetic muscle strength during resistance exercises with varying loads and protocols. Furthermore, lower doses of caffeine (2–3 mg/kg) were found to offer similar enhancement effects with high doses of caffeine (e.g., 6 mg/kg)^25^. In a review published by the International Society of Sports Nutrition (ISSN) in 2021, it was stated that the effective minimum dose of caffeine may be 2 mg/kg^26^. Although the dose in the present study was only half of the previous study, it still had the effect of enhancing athletic performance, which suggests that the lower dose of caffeinated chewing gum (2.75 ± 0.53 mg/kg) in this study, there may be positive effects on muscle performance during flywheel exercise. This observation aligns with emerging research suggesting that caffeine's impact on performance may extend to lower dosage ranges. The rationale for investigating lower doses is rooted in the pursuit of optimizing sports performance while considering athlete safety and anti-doping regulations. It's important to note that individual responses to caffeine can vary due to genetic factors, tolerance levels, and other variables. Therefore, future research in this area is essential to better understand the mechanisms behind the potential benefits of low-dose caffeine supplementation and its practical implications for athletes.

Even though, the effect of caffeinated chewing gum on the performance of the Romanian deadlift on a flywheel is still unclear. The flywheel training device is a method specifically designed for eccentric training^27^. Studies have shown that eccentric training can lead to greater improvements in strength, eccentric force and muscle mass in healthy adults compared to concentric training^6,28^. In a study by Rønnestad et al., an additional 12 weeks of heavy weight training was found to increase peak power output on the Wingate test in cyclists, as well as increase muscle cross-sectional area in knee extension and flexion and maximum isometric strength in the half squat^29^. In another study by Beattie et al., after 20 weeks of maximal strength and explosive training, results were found to increase maximal strength and specific explosive power as well as muscle mass in the lower extremities of cyclists^30^. As found in the above mentioned literature, the power output and muscle cross-sectional area of the lower extremities can be increased after high intensity resistance training, which means that it may increase maximal muscle strength or muscle mass, and also improve power output for specific sports. When muscle power output and muscle volume are improved, the state of muscle work will also increase, because the work will be proportional to the product of force output and displacement, so the quality of training and the rate of muscle growth will also be improved. It offers athletes and coaches the possibility to consider the use of caffeinated chewing gum during training with the flywheel training device to enhance the training effect.

## Strength and limitations

This trial used a rigorous two-arm crossover randomized controlled design. All outcomes were measured by Exxentric kMeter (© Exxentric AB, Sweden) to ensure reliability^14^. Despite these strengths, the study has several limitations. The main limitation is that the study design is single-blind. Although the researcher who analyzed the data was not blinded, they did not pay attention to which group the participants were in and did not encourage them during the exercise. Besides, the study results were provided by the Exxentric kMeter. By using device-based measurements, this study ensured that the outcome measures accurately reflected the participants' performance. In addition, we did not detect experimental conditions about caffeine they consumed on the various visits. However, we controlled the food diary for 24 h during both experimental visits and avoided food and drinks with caffeine during this period. This study did not include a baseline measurement of RDL performance without the administration of caffeine (CAF) or placebo (PL), which could have served as a valuable comparison to better understand the effects of the interventions. Moreover, the effectiveness of blinding might have been influenced by the participants' beliefs regarding which intervention they received (CAF or PL). These factors could potentially affect the outcomes and interpretations of the study, and should be considered when evaluating the results.

This study provides new evidence on the potential benefits of using caffeinated chewing gum to enhance physical performance. However, it is important to note that the field is still in its infancy and our understanding is largely based on limited research. Therefore, there is an urgent need for larger clinical trials to robustly determine the efficacy and safety of caffeinated chewing gum in improving physical performance. Future research should focus on different doses, timing of ingestion, and interactions of caffeine with different physical activities. Finally, we used absolute rather than relative doses of caffeine due to logistical constraints associated with individual chewing gum formulations. This approach may not accurately reflect real-world situations where caffeine intake may vary according to personal response.

## Conclusions

We investigated the effect of a caffeinated chewing gum supplementation on the performance of the Romanian deadlift on a flywheel training device. Caffeine supplementation via caffeinated chewing gum at a dose of 200 mg for 10 min, taken 15 min prior to the Romanian deadlift on the flywheel training device, improved peak concentric power, peak eccentric power, average power, and total work performance compared to placebo. However, the two trials had no differences in average force, rating perceived exertion, and heart rate. This allows athletes and coaches to consider using caffeinated chewing gum during training with the flywheel trainer to enhance the training effect.

## Data Availability

All relevant materials are presented in the present manuscript.
