# Author Correction: Acute enhancement of Romanian deadlift performance after consumption of caffeinated chewing gum

**DOI:** 10.1038/s41598-024-57126-7

**Published:** 2024-03-19

**Authors:** Chun-Hung Chen, Shih-Hao Wu, Yi-Jie Shiu, Sheng-Yan Yu, Chih-Hui Chiu

**Affiliations:** 1 Department of Emergency Medicine, China Medical University Hospital, China Medical University, Taichung, Taiwan; 2 College of Medicine, China Medical University, Taichung, 404 Taiwan; 3 Division of Toxicology, China Medical University Hospital, China Medical University, Taichung, Taiwan; 4 Graduate Program in Department of Exercise Health Science, National Taiwan University of Sport, No. 16, Sec. 1, Shuang-Shih Rd., Taichung, 404 Taiwan; 5 Department of Physical Education and Sport Sciences, National Taiwan Normal University, Taipei, Taiwan

Correction to: *Scientific Reports* 10.1038/s41598-023-49453-y, published online 12 December 2023

In the original version of this Chun-Hung Chen and Shih-Hao Wu were omitted as equally contributing authors.

The original Article has been corrected.

